# Peripheral arterial oxygen saturation to fraction of inspired oxygen ratio: a versatile parameter for critically ill patients

**DOI:** 10.62675/2965-2774.20250156

**Published:** 2025-01-15

**Authors:** Eduardo Butturini de Carvalho, Bruno Valle Pinheiro, Pedro Leme Silva

**Affiliations:** 1 Universidade de Vassouras Vassouras RJ Brazil Universidade de Vassouras - Vassouras (RJ), Brazil.; 2 Pulmonary and Critical Care Division Hospital Universitário Universidade Federal de Juiz de Fora Juiz de Fora MG Brazil Pulmonary and Critical Care Division, Hospital Universitário, Universidade Federal de Juiz de Fora - Juiz de Fora (MG), Brazil.; 3 Laboratory of Pulmonary Investigation Instituto de Biofísica Carlos Chagas Filho Universidade Federal do Rio de Janeiro Rio de Janeiro RJ Brazil Laboratory of Pulmonary Investigation, Instituto de Biofísica Carlos Chagas Filho, Universidade Federal do Rio de Janeiro - Rio de Janeiro (RJ), Brazil.

Acute hypoxemic respiratory failure (AHRF) is a highly prevalent condition in critically ill patients. Regardless of the different causes leading to AHRF, a cornerstone diagnostic tool is calculating PaO_2_/FiO_2_ from arterial blood gas analysis. Nevertheless, PaO_2_/FiO_2_ has several practical limitations: arterial puncture is a painful procedure^1^ that is technically challenging in some patients, and blood gas analyzers might not be widely available in prehospital care and hospitals in low-income and lower-middle-income countries. Because of these limitations, several authors have proposed the replacement of PaO_2_/FiO_2_ with SpO_2_/FiO_2_ as a tool not only for hypoxemia diagnosis but also for monitoring and determining prognosis.^2-7^Pulse oximeters are a low-cost, noninvasive technology widely used in healthcare. They determine oxygen saturation by measuring the light absorption of arterial blood at two specific wavelengths, namely, 660nm (red) and 940nm (infrared). The relative absorption at these two wavelengths, calibrated against direct measurements of SaO_2_, generates the pulse-estimated SpO_2_.^8^Pulse oximetry is accurate in reflecting SaO_2_ when its value is above 90%. However, the accuracy worsens when the SaO_2_ is lower than 90%, systematically underestimating SaO_2_ when it is 80% or less.^9^

**Figure 1:**
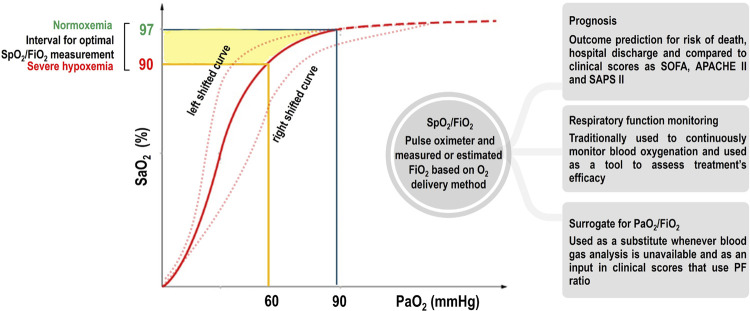
Oxyhemoglobin dissociation curve. In the flat portion of the curve (dashed line), changes in arterial oxygen partial pressure produce small or no changes in arterial oxygen saturation (when values are greater than 97%).

The oxyhemoglobin dissociation curve, from which SpO_2_ is derived, is based on a well-established physiological concept, and its comprehension is important to understand some limitations in adopting SpO_2_ and SpO_2_/FiO_2_ as markers of PaO_2_/FiO_2_ in clinical practice. The oxyhemoglobin dissociation curve has a sigmoidal shape, with a flat upper portion (Figure 1). In this flat portion, significant changes in the PaO_2_ produce only small changes in SpO_2,_ and the relationship between SpO_2_ and PaO_2_ dramatically decreases. Therefore, to use SpO_2_/FiO_2_ for AHRF diagnosis and monitoring, SpO_2_ must be ≤ 97%.^10^ In contrast, measuring SpO_2_/FiO_2_ in room air may also be an issue since patients with AHRF may develop life-threatening hypoxemia, and SpO_2_/FiO_2_ may lack sensitivity for AHRF diagnosis.^11^ Moreover, several factors can shift the oxyhemoglobin dissociation curve to the left or to the right, changing the correlations between SpO_2_ and PaO_2_. The impact of these possible shifts in the accuracy of SpO_2_/FiO_2_ has not yet been well established (Figure 1).

When the curve is left shifted (due to, for example, alkalosis, low body temperature, low 2,3-diphosphoglycerate, or decreased PaCO_2_), SaO_2_ overestimates PaO_2_. When the curve is right shifted (due to, for example, acidosis, high body temperature, high 2,3-diphosphoglycerate, or increased PaCO_2_), SaO_2_ underestimates PaO_2_. The interval between normoxemia (at 97% SaO_2_) and hypoxemia (at 90% SaO_2_) represents the optimal range for SpO_2_/FiO_2_ measurement, which comprises intervals of PaO_2_ of 90 and 60mmHg, respectively.^(^^5^^,^^12^^)^

Since the first attempts to replace PaO_2_/FiO_2_ for SpO_2_/FiO_2_, the SpO_2_/FiO_2_ ratio has been shown to be a promising tool in different scenarios. First, it has good performance as a prognostic variable. A 2020 COVID-19 cohort revealed a strong association between SpO_2_/FiO_2_ and the risk of death.^5^ Similar results were obtained in AHRF patients in the intensive care unit (ICU),^12^ even showing a better mortality prediction ability for SpO_2_/FiO_2_ than well-established clinical scores such as the SOFA, APACHE II, and SAPS II. Using artificial intelligence, researchers identified SpO_2_/FiO_2_ as an independent predictor of ICU (OR = 2.73) and day-28 survival (OR = 3.96).^13^ Second, it has been successfully used to monitor respiratory function and assess treatment efficacy; for example, preterm infants in whom surfactant treatment failed had lower SpO_2_/FiO_2_.^14^ Third, it has been used as a surrogate for PaO_2_/FiO_2_ in AHRF diagnosis and as an input for PaO_2_/FiO_2_ in clinical scores. A SpO_2_/FiO_2_ ratio threshold of 350 had a positive predictive value of 0.88 for a PaO_2_/FiO_2_ < 300, and a SpO_2_/FiO_2_ threshold of 470 had a negative predictive value of 0.89 for a PaO_2_/FiO_2_ < 400. In a study with 703 critically ill patients, nonlinear PaO_2_/FiO_2_ imputation from SpO_2_/FiO_2_ had 0.9 sensitivity and 0.67 specificity for a PaO_2_/FiO_2_ < 300.^15^ More recently, the New Global Definition for Acute Respiratory Distress Syndrome^16^ allowed the use of SpO_2_/FiO_2_ ≤ 315 (if SpO_2_ ≤ 97%) as an alternative to PaO_2_/FiO_2_ ≤ 300mmHg for the diagnosis of the syndrome. SpO_2_/FiO_2_ can also be used for classifying acute respiratory distress syndrome as mild (235 < SpO_2_/FiO_2_ ≤ 315), moderate (148 < SpO_2_/FiO_2_ ≤ 235) or severe (SpO_2_/FiO_2_ ≤ 148).

SpO_2_/FiO_2_ still has some important limitations, other than those related to oxyhemoglobin dissociation curve characteristics, to directly replace PaO_2_/FiO_2_ in clinical practice. First, their relationship is not perfectly linear, although studies have found regression equations that can reasonably predict PaO_2_/FiO_2_ from SpO_2_/FiO_2_.^4^ Second, SpO_2_ reading errors can occur due to hypoperfusion, hypothermia, malposition of the probe, racial differences,^17^ or motion artifacts. Third, several conditions may lead to falsely elevated readings (i.e., carboxyhemoglobin, methemoglobin, sulfhemoglobin, or skin pigment) or falsely low readings (i.e., severe anemia, sickle hemoglobin, methemoglobin, sulfhemoglobin, nail polish, or vital dyes).^18^ Finally, the SpO_2_/FiO_2_ ratio logically depends on the FiO_2_ amount, which can be challenging to measure precisely in some scenarios, especially with conventional oxygen therapy, nasal cannulas, and face masks with or without reservoirs, although conversion tables are available (Table 1S - Supplementary Material). However, this limitation also occurs when PaO_2_/FiO_2_ is used.

Future clinical studies addressing SpO_2_/FiO_2_ should not only try to estimate PaO_2_/FiO_2_ on the basis of an equation but also attempt to establish cutoff values and prognostic values for SpO_2_/FiO_2_ under specific protocols for mechanically ventilated or spontaneous breathing patients, defining *a priori* an optimal FiO_2_ range. Strategies such as the SpO_2_/FiO_2_ diagram,^11^ constructed by decremental FiO_2_ titration from 100% to 21%, could be tested in an attempt to increase the specificity and specificity values for AHRF diagnosis. Thus far, it may be too early to recommend SpO_2_/FiO_2_ as a perfect replacement for PaO_2_/FiO_2_, since strong evidence from prospective studies is still lacking. Nevertheless, it is not just a promising tool but rather an option for diagnosis, monitoring, and prognosis when arterial blood gas analysis is not readily available or when its risks outweigh its benefits.
